# False-negative frozen section of sentinel nodes in early breast cancer (cT1-2N0) patients

**DOI:** 10.1186/s12957-021-02288-1

**Published:** 2021-06-22

**Authors:** Zhu-Jun Loh, Kuo-Ting Lee, Ya-Ping Chen, Yao-Lung Kuo, Wei-Pang Chung, Ya-Ting Hsu, Chien-Chang Huang, Hui-Ping Hsu

**Affiliations:** 1 Department of Surgery, National Cheng Kung University Hospital, College of Medicine, National Cheng Kung University, Tainan, Taiwan; 2 Division of Hematology, Department of Internal Medicine, National Cheng Kung University Hospital, College of Medicine, National Cheng Kung University, Tainan, Taiwan; 3 Department of Oncology, National Cheng Kung University Hospital, College of Medicine, National Cheng Kung University, Tainan, Taiwan; 4 Institute of Clinical Medicine, College of Medicine, National Cheng Kung University, Tainan, Taiwan; 5 Center of Applied Nanomedicine, National Cheng Kung University, Tainan, Taiwan; 6 Department of Biostatistics, Vanderbilt University Medical Center, Nashville, USA

**Keywords:** Early breast cancer, Sentinel lymph node biopsy, False-negative frozen section, Sentinel nodes, Axillary lymph node dissection

## Abstract

**Background:**

Sentinel lymph node biopsy (SLNB) is the standard approach for the axillary region in early breast cancer patients with clinically negative nodes. The present study investigated patients with false-negative sentinel nodes in intraoperative frozen sections (FNSN) using real-world data.

**Methods:**

A case–control study with a 1:3 ratio was conducted. FNSN was determined when sentinel nodes (SNs) were negative in frozen sections but positive for metastasis in formalin-fixed paraffin-embedded (FFPE) sections. The control was defined as having no metastasis of SNs in both frozen and FFPE sections.

**Results:**

A total of 20 FNSN cases and 60 matched controls from 333 SLNB patients were enrolled between April 1, 2005, and November 31, 2009. The demographics and intrinsic subtypes of breast cancer were similar between the FNSN and control groups. The FNSN patients had larger tumor sizes on preoperative mammography (*P* = 0.033) and more lymphatic tumor emboli on core biopsy (*P* < 0.001). Four FNSN patients had metastasis in nonrelevant SNs. Another 16 FNSN patients had benign lymphoid hyperplasia of SNs in frozen sections and metastasis in the same SNs from FFPE sections. Micrometastasis was detected in seven of 16 patients, and metastases in nonrelevant SNs were recognized in two patients. All FNSN patients underwent a second operation with axillary lymph node dissection (ALND). After a median follow-up of 143 months, no FNSN patients developed breast cancer recurrence. The disease-free survival, breast cancer-specific survival, and overall survival in FNSN were not inferior to those in controls.

**Conclusions:**

Patients with a larger tumor size and more lymphatic tumor emboli have a higher incidence of FNSN. However, the outcomes of FNSN patients after completing ALND were noninferior to those without SN metastasis. ALND provides a correct staging for patients with metastasis in nonsentinel axillary lymph nodes.

## Background

In Taiwan, breast cancer is the most common malignancy in women, with the incidence reaching a plateau at approximately 50 years of age and the prevalence increasing in all age groups in recent years [1, 2]. With the increase in the prevalence of lymphatic spread of breast cancer, standardized operation with radical mastectomy and axillary lymph node dissection (ALND) was established by Dr. Halsted in 1894 [3], and since the development of adjuvant therapy, many patients survive after radical resection. However, after ALND, survivors may have severe lymphedema over their ipsilateral upper limb [4]. The sentinel lymph node biopsy (SLNB) technique was developed to reduce the number of resected lymph nodes in patients with clinically negative nodes [5]. The clinical trial ACOSOG Z0011 was conducted in 889 patients with cT1-2N0 breast cancer and one to two positive sentinel nodes (SNs) treated by breast-conserving therapy (BCT) with SLNB or ALND. The results revealed no difference in nodal recurrence, axillary failure, or patient survival. ALND can be avoided in early breast cancer patients with one to two positive SNs and receiving BCT and replaced by adjuvant whole-breast irradiation [6–8]. However, the ACOSOG Z0011 trial did not enroll patients with large tumors or those who received a total mastectomy. SLNB is also not appropriate for patients with positive preoperative node biopsies [9]. Furthermore, the indication of ALND in patients undergoing total mastectomy should be considered.

The sensitivity and specificity of SLNB are the first considerations. Mapping of axillary lymph nodes helps to advance the correctness of SLNB [10], and localization of SNs by two kinds of indicators is a standard procedure. However, intraoperative evaluation of SNs is diverse. Some institutes use cytological analysis of touch imprints, whereas others accept frozen sections of the SN [11, 12]. Intraoperative evaluation of SNs detects metastasis, for which the operation is immediately changed to ALND. Overall, intraoperative frozen section analysis of SNs has a sensitivity of 87% and a specificity of 100% [12]. Unfortunately, 3% of patients are recalled for ALND because of a false-negative result of the frozen section during the first operation [12]. The number of SNs is also a confounding factor for false-negative results of SLNB, and harvesting only one SN contributes to a high rate of axillary recurrence [13]. The long-term clinical outcomes of patients with false-negative SNs are the second consideration. For patients who have micrometastasis (metastatic size greater than 0.2 mm and equal or less than 2 mm) in the SNs, the hazard ratio of regional recurrence is 4.39 for those undergoing SLNB alone compared with those receiving ALND [14]. Other clinical studies have concluded that the incidence of regional recurrence is very low in breast cancer patients with SN micrometastasis [15, 16]. For patients who have macrometastasis (metastatic size greater than 2 mm) in the SNs, avoiding ALND will result in the underestimation of lymph node status and may lead to cancer mis-staging [17]. Patients with lymph node metastasis also have a higher incidence of recurrence in the ipsilateral regional lymph nodes [18]. In the present study, we investigated the predisposing factors for false-negative frozen sections of SNs in patients with breast cancer and clinically negative nodes. We randomly enrolled patients planning to receive partial or total mastectomy using real-world data. This study also aimed to evaluate the long-term outcome of patients with false-negative frozen sections of SNs.

## Patients and methods

The study was designed as a case–control study with a case-to-control ratio of 1:3. Patients were diagnosed with invasive ductal carcinoma of the breast and recruited between April 1, 2005, and November 31, 2009. All patients had clinically negative axillary or internal mammary lymph nodes. The surgical planning included SLNB. The operative method for primary breast cancer was decided by the attending surgeon and the patient after a thorough discussion. Patients with ductal or lobular carcinoma in situ, metastatic disease, clinically node-positive disease, inflammatory breast cancer, and other variants of carcinoma (lobular, mucinous, metaplastic carcinomas, phyllodes, sarcoma, or lymphoma) and those who had received neoadjuvant chemotherapy, previous axillary surgery, or radiation were excluded. Pathological stage was classified according to the criteria defined by the American Joint Committee on Cancer (AJCC) Staging Manual, 7th edition. The study was reviewed and monitored by the Institutional Review Board of National Cheng Kung University Hospital (A-ER-105-233). Intrinsic subtypes were defined by immunohistochemistry staining of estrogen receptor (ER), progesterone receptor (PR), and human epidermal growth factor type II receptor (HER2).

### Identification of sentinel nodes

All clinical information was obtained by retrospective chart review. Dual methods were used to identify SNs. All patients received a peritumoral subcutaneous injection of 1 mCi Technetium-99m phytate (Fujifilm RI Pharma, Chiba, Japan), after which a lymphoscintigraphy series was performed. SNs with radioactive signals were identified by a handheld probe of the Navigator System (RMD, Watertown, MA, USA) during the operation. After anesthesia, 2 mL of methyl blue was injected around the tumor in the subcutaneous layer. Gentle massage for 5 min was performed from the tumor to the ipsilateral axillary region to facilitate the transmission of methyl blue along the lymphatic ducts. Relevant SNs were defined as blue-stained nodes with ex vivo radioactive counts of at least 10% in situ counts. If a node was only blue-stained or only had high radioactive counts, it was defined as a nonrelevant SN. The number of relevant SNs sent for frozen section analysis was decided by the attending surgeon.

Intraoperative frozen sections were standardized. SNs of more than 4 mm were bisected along their long axis. Half of the nodes were embedded in an optimal cutting temperature compound (OCT) (Sakura Finetec, Torrance, CA, USA) and frozen in a − 20 °C refrigerator. Small sentinel lymph nodes (less than or equal to 4 mm) were completely frozen. Sections of 4 mm were cut and stained with hematoxylin and eosin (H&E) for frozen examination. After the preliminary frozen diagnosis, the OCT-embedded tissue of all nodes and the other half of the large nodes were fixed in formalin. Three levels of permanent sections were taken from formalin-fixed paraffin-embedded (FFPE) blocks. The final diagnosis of sentinel lymph nodes was based on permanent sections.

### Enrollment of patients

Case subjects (the false-negative frozen section of sentinel node [FNSN] group) included patients who were diagnosed as being negative for lymph node metastasis by frozen examination but turned were positive for metastasis in permanent sections from FFPE samples. Potential case subjects were identified from the registry list at the Cancer Center in National Cheng Kung University Hospital. Two chart reviewers examined the registry information. Three control subjects per case subject were selected from the same registry list and matched with the case subjects according to age at diagnosis (within 2 years), date of operation (within 1 year), tumor stage, and intrinsic subtype (ER/PR/HER2).

All patients received preoperative examinations with sonography or mammography. The characteristics of the breast tumors were recorded, and the report was categorized according to the Breast Imaging Reporting and Data System (BI-RADS) [19]. Preoperative diagnosis of breast cancer was according to the cytological study of fine-needle aspiration (FNA) or pathological examination of core needle biopsy (CNB). Some patients received preoperative examinations in other hospitals, resulting in missing data.

### Statistical analysis

Differences between the case and control groups were compared using a chi-square test or Fisher’s exact test for categorical variables. Continuous variables were analyzed by the nonparametric Mann–Whitney test. Multivariate analysis using the logistic regression model was applied to determine significant predictors of FNSN. Each model included age as a covariate, and the results are expressed as odds ratios (ORs) with 95% confidence intervals (CIs). Survival curves were drawn using the Kaplan–Meier method, and group differences in survival time were calculated by a log-rank test. The definition of breast cancer-related disease-free survival (DFS) was the time from the date of the first operation to the date of the first recurrence of breast cancer or death from any other cause. Breast cancer recurrence was defined as an event, and cases of death due to any other cause were censored. Overall survival (OS) was defined as the time from the date of the first operation to the date of death from any cause. Breast cancer-specific survival (BCSS) was the time from the date of the first operation to the date of death from breast cancer. Patients who died because of other causes were censored while calculating BCSS. The Cox proportional hazard model for survival was applied for hazard ratios (HRs) and 95% CIs. All statistical tests were conducted in SPSS version 17.0 (IBM, SPSS Inc., Chicago, IL, USA), and a *P* value of less than 0.05 was defined as statistically significant.

## Results

### Patient characteristics

Between April 1, 2005, and November 31, 2009, 1810 patients were diagnosed with breast cancer in our hospital. Of these, 1525 received surgical intervention, whereas 333 underwent SLNB. There were 20 patients with invasive ductal carcinoma and false-negative results by frozen section for SLNB, defined as the FNSN group. Matched controls were selected from among those with invasive ductal carcinoma who underwent SLNB with true-negative results based on frozen sections. Demographics, tumor characteristics, and operative procedures were similar between FNSN and controls (Table 1).

**Table 1:** Demographic and tumor characteristics of subjects with a false-negative frozen section of sentinel nodes (FNSN) and controls

	Control (*n* = 60)	FNSN (*n* = 20)	*P* value
Age	48 (35–73)	48 (37–71)	0.772
Operative method for breast cancer			0.599
Total mastectomy	22 (37%)	9 (45%)	
Partial mastectomy	38 (63%)	11 (55%)	
Tumor size, cm, median (range)	1.7 (0.3–3.2)	1.8 (1.0–4.5)	0.331
Tumor stage			> 0.999
T1	43 (72%)	14 (70%)	
T2	17 (28%)	6 (30%)	
Histological grade			0.418
Grade I	18 (30%)	3 (15%)	
Grade II	20 (33%)	8 (40%)	
Grade III	22 (37%)	9 (45%)	
Extensive intraductal component	21 (35%)	5 (25%)	0.582
Fascia invasion	0	2 (10%)	0.060
Skin invasion	0	1 (5%)	0.250
Nipple invasion	0	2 (20%)	0.103
Estrogen receptor			0.750
Negative	12 (20%)	3 (15%)	
Positive	48 (80%)	17 (85%)	
Progesterone receptor			0.433
Negative	25 (42%)	6 (30%)	
Positive	35 (58%)	14 (70%)	
HER2/Neu (Immunohistochemical staining)			> 0.999
Negative	52 (87%)	18 (90%)	
Positive	8 (13%)	2 (10%)	
Intrinsic subtypes			0.761
HmR-positive, HER2-negative	43 (72%)	16 (80%)	
HER2-enriched	8 (13%)	2 (10%)	
TNBC	9 (15%)	2 (10%)	

### Preoperative assessment

**Fig. 1:**
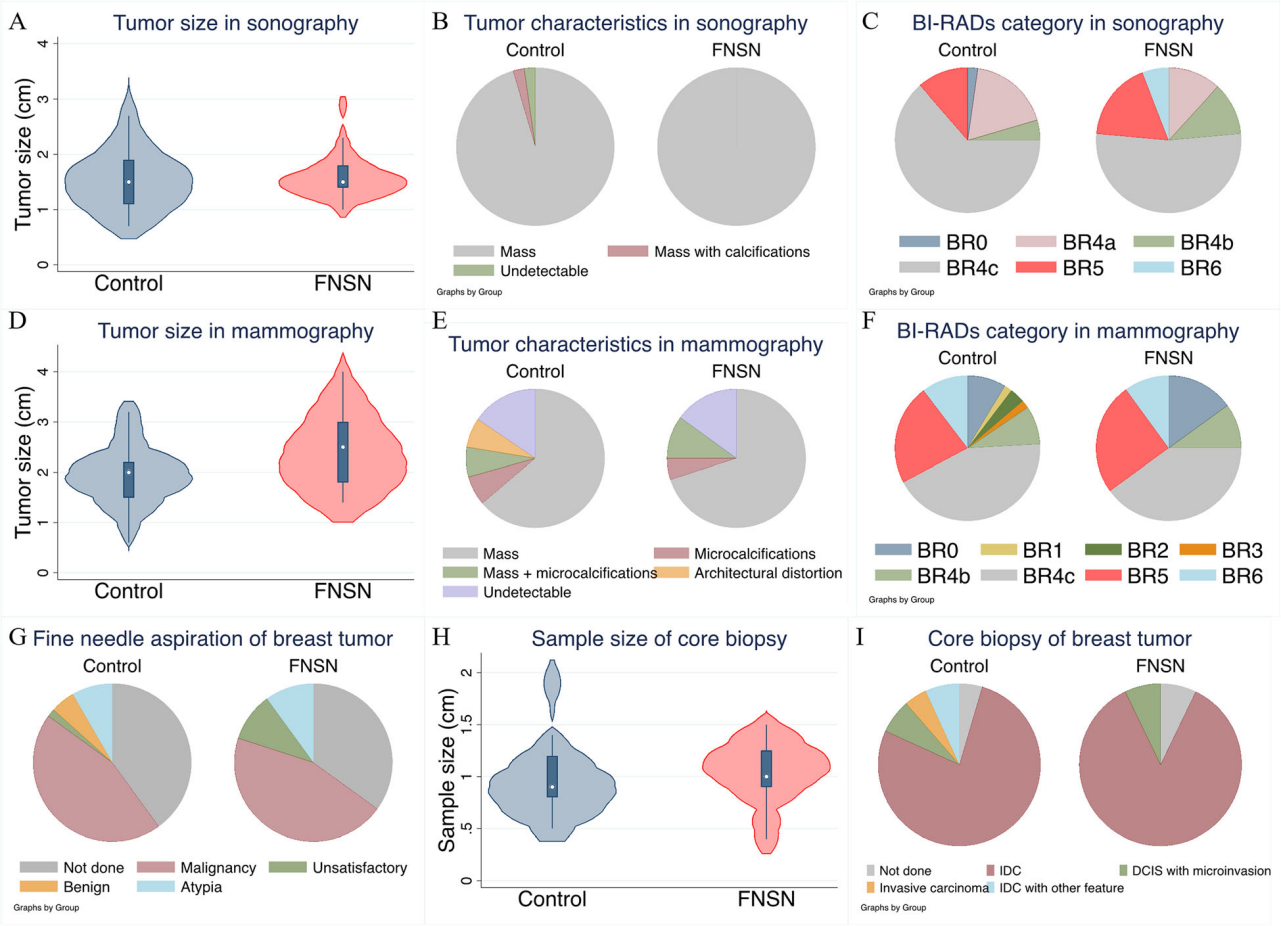
Preoperative imaging studies. **A** Size of detectable tumors on sonography. **B** Tumor characteristics on sonography. **C** Sonography results were categorized by Breast Imaging Reporting and Data System (BI-RADS). The tumor shown was not identified by sonography, or patients who did not receive sonography in our hospital were excluded from these three analyses. **D** Size of detectable masses by mammography. The area of microcalcifications and architectural distortion were not included in the measurement of the tumor size. **E** Characteristics of breast cancer by mammography. **F** Results of mammography were categorized by BI-RADS. The tumor failed to be identified by mammography, and patients who did not receive mammography in our hospital were excluded from these three analyses. **G** Cytology results of fine-needle aspiration of a primary breast tumor. **H** Long-axis length of samples from core needle biopsy. **I** Histological results of core needle biopsy for a primary breast tumor. Patients who did not receive tissue-proven examinations in our hospital were excluded from these analyses

In total, 61 patients received sonography, and 78 underwent diagnostic mammography in our hospital. Sonography failed to detect the tumor in one patient in the control group. Tumor characteristics and sizes of primary breast cancer were similar between the FNSN and control groups (Fig. 1A, B), and sonography results did not differ (Fig. 1C). However, FNSN patients had a larger breast tumor size on mammography (Fig. 1D, *P* = 0.033). Diagnostic mammography was unable to detect abnormalities in 15% of FNSN patients and 16% of controls (Fig. 1E), and categorical results of BI-RADS were similar in these two groups (Fig. 1F).

There were 36 patients in the control group and 13 in the FNSN group who received FNA of the primary breast tumor. Three patients (two in the control and one in FNSN) in the present study only received FNA preoperatively (Fig. 1G). The gold standard for preoperative diagnosis of breast cancer is the pathological examination of CNB, which 77 patients received preoperatively. A total of 42 patients in the control group and 13 in the FNSN group received CNB in our hospital, and the cumulative size of the samples in CNB was equal between the two groups (Fig. 1H). Three patients in the control group and one in the FNSN group were diagnosed with ductal carcinoma in situ with microinvasion in CNB. Two patients in the control group were diagnosed with invasive carcinoma without specific types, and three had other features of carcinomas. All these patients were confirmed to have invasive ductal carcinoma in the final pathological examination (Fig. 1I).

### Frozen section of sentinel nodes

All patients received localization of the sentinel lymph nodes by dual methods. Relevant SNs were sent for frozen sectioning; other SNs were preserved in FFPE blocks and included in the final reports. The frozen sections of relevant SNs from four patients in the FNSN group were negative for malignancy in the final pathological report of FFPE blocks; however, metastatic foci were detected in nonrelevant SNs. For 16 other FNSN patients, the results were benign lymphoid hyperplasia based on SN frozen sections, whereas the final pathological report for FFPE blocks of SNs was metastatic carcinoma (Table 2). The correct evaluation of the pathological stage was performed after the second operation with ALND in the FNSN patients, and these patients exhibited a trend of a greater number of total SNs during the first operation (*P* = 0.072, Table 2). The number of resected and positive lymph nodes was higher in the FNSN group than in the control group, as all FNSN patients underwent a second operation with ALND. The patients in the FNSN group also had a higher proportion of lymphatic tumor emboli (LTE) in CNB and an advanced nodal or AJCC tumor–node–metastasis stage in the final reports (Table 2). Multivariate analysis of age, tumor size in mammography, LTE, and number of total SNs was conducted, and only LTE showed significant predictive power for FNSN (Table 3).

**Table 2:** Pathologic nodal findings from intraoperative analysis and final diagnosis of subjects with a false-negative frozen section of sentinel nodes (FNSN) and controls

	Control (n = 60)	FNSN (n = 20)		*P* value
Frozen section of lymph nodes				< 0.001
Frozen section for relevant SNs and metastasis in nonrelevant SNs	0	4 (20%)		
False-negative results	0	16 (80%)		
Micrometastasis in SNs			7	
Metastasis in SNs and nonsentinel ALNs			1	
Metastasis only in SNs			6	
Macrometastasis in SNs			9	
Metastasis in SNs and nonsentinel ALNs			1	
Metastasis only in SNs			8	
True-negative results	60 (100%)	0		
Lymphatic tumor emboli	10 (17%)	18 (90%)		< 0.001
Number of relevant SNs for frozen section	1 (0–9)	2 (1–4)		0.881
Number of hot spots in lymphoscintigraphy	1 (1–5)	1 (1–4)		0.570
Total number of SNs during the 1st operation	3 (1–13)	5.5 (1–17)		0.072
Lymph node invasion				< 0.001
Negative	60 (100%)	0		
Positive	0	20 (100%)		
Resected lymph nodes (including SLNB and ALND)	3 (1–13)	23 (9–39)		< 0.001
Positive lymph nodes (including SLNB and ALND)	0	2 (1–7)		< 0.001
Nodal stage				< 0.001
N0	60 (100%)	0		
N1	0	17 (85%)		
N2	0	3 (15%)		
AJCC TNM stage				< 0.001
Stage IA	43 (72%)	0		
Stage IIA	17 (28%)	12 (60%)		
Stage IIB	0	5 (25%)		
Stage IIIA	0	3 (15%)

**Table 3:** Multivariate analysis of predictors for FNSN in breast cancer patients

		HR	95% CI	*P* value
Age		1.03	0.943–1.129	0.499
Lymphatic tumor emboli	Negative	1.0		
	Positive	47.56	9.23–245.04	< 0.001

### Long-term survival

**Fig. 2:**
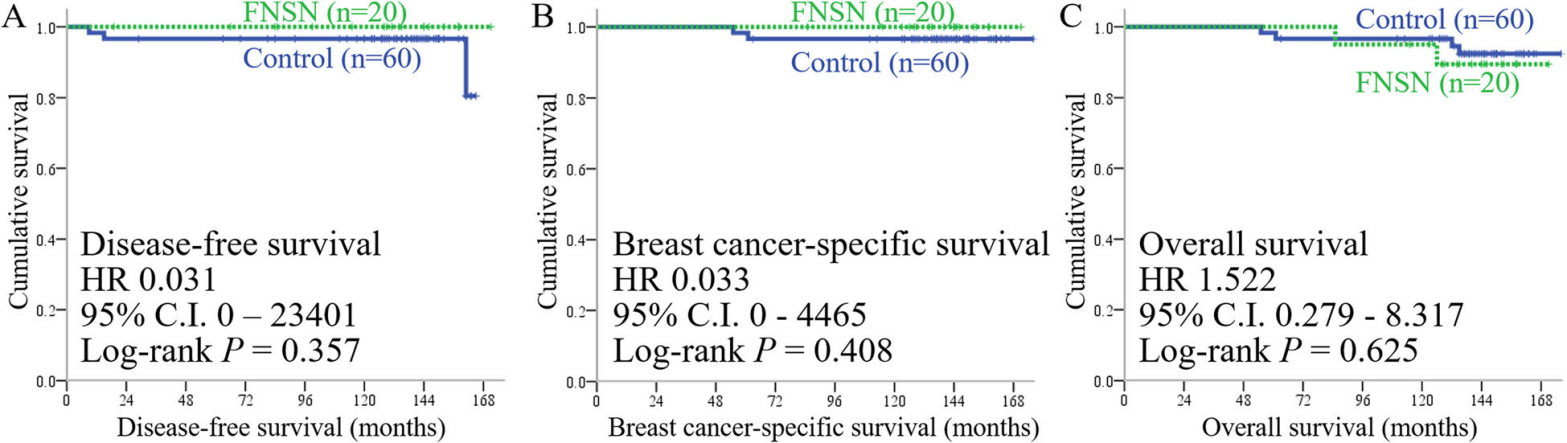
Kaplan–Meier survival analysis. **A** Breast cancer-related disease-free survival, **B** breast cancer-specific survival, and **C** overall survival in the present study, with the hazard ratio (HR), 95% confidence interval (CI), and *P* value from the log-rank test

The median follow-up time was 143 months for all patients, with a range of 55 to 176 months. All patients received standardized adjuvant therapy designed by the attending physicians. Three patients in the control group developed recurrence during follow-up, and all of them received salvage therapy, with two breast cancer-related mortalities (Table 4). No patient in the FNSN group developed recurrence. DFS (HR 0.031, 95% CI 0–23,401 in Fig. 2A), BCSS (HR 0.033, 95% CI 0–4465 in Fig. 2B), and OS (HR 1.522, 95% CI 0.279–8.317 in Fig. 2C) were similar between the FNSN and control groups.

**Table 4:** Disease-free survival events and number of deaths among subjects with a false-negative frozen section of sentinel nodes (FNSN) and controls

	Control (*n* = 60)	FNSN (*n* = 20)	*P* value
Disease-free survival events*	54	18	> 0.999
Breast cancer metastasis events	3 (5%)	0	0.569
Lung	2 (3%)	0	> 0.999
Liver	2 (3%)	0	> 0.999
Bone	3 (5%)	0	0.569
Brain	1 (2%)	0	> 0.999
Regional lymph nodes (axillary and/or internal mammary chains)	1 (2%)	0	> 0.999
Distant lymph nodes	2 (3%)	0	> 0.999
Local recurrence	1 (2%)	0	> 0.999
Nonbreast cancer deaths	3 (5%)	2 (10%)	0.594
Other malignancies	2 (3%)	1 (5%)	> 0.999
Sepsis	1 (2%)	0	> 0.999
Unknown	0	1 (5%)	0.250
Breast cancer-related deaths	2 (3%)	0	> 0.999

*Excluding breast cancer-related and nonbreast cancer-related events

## Discussion

The standard operation for patients with early-stage breast cancer and clinically negative nodes includes total or partial mastectomy and SLNB. In the present study, we evaluated the FNSN of the frozen sections during the first operation. A case-matched control study was designed with a one-to-three ratio. The FNSN patients had a larger tumor size in preoperative mammography and an increased ratio of LTE in CNB. These patients also had a greater number of SNs during the first operation. However, LTE was the only predictive variable for FNSN in the multivariate analysis. Metastasis was detected in nonrelevant SNs from four FNSN patients and in relevant SNs based on FFPE sections for 16 patients, which was not found during the intraoperative frozen section analysis. Seven FNSN patients had micrometastasis in relevant SNs. Two FNSN patients had metastasis in relevant SNs and nonsentinel axillary lymph nodes (ALNs). All 20 FNSN patients underwent a second operation with ALND. Long-term outcomes of the FNSN and control groups were similar, with no difference in DFS, BCSS, or OS. Moreover, no breast cancer-associated events occurred in the FNSN groups.

Some investigators advise that intraoperative assessment be reserved for patients with clinically positive nodes or those with aggressive diseases after neoadjuvant chemotherapy [20], and one reason is the cost of frozen sections. Nonetheless, studies still recommend intraoperative assessment of SNs. For breast cancer with clinically negative nodes, intraoperative frozen section analysis has a sensitivity of 87%, a specificity of 100%, and a patient recall rate of 3% [12]. Risk factors for FNSN include tumor location, lymphovascular invasion, suspicious nodes in the preoperative study, less than three SNs, larger tumor size, invasive lobular carcinoma, hormone receptor-negative cancer, and poorly differentiated cancer [21–23]. The FNSN rate ranges from 3.1 to 18% in the literature [21, 23]. In the present study, we excluded patients with invasive lobular carcinoma because of the difficulty in diagnosing these samples by H&E staining. There were 20 patients with FNSN among 333 SLNB patients. The proportion of FNSN is 6.0% in our hospital. A larger tumor size by preoperative mammography and LTE in the samples of CNB were found to be associated with an increased risk of FNSN (Table 2), and the patients with FNSN had a greater number of total SNs during the first operation. In the case of LTE in a CNB specimen, a tumor larger than 2.5 cm on preoperative mammography, and high residual radioactivity over the axillary region during the operation, the patient and family should be informed of the higher risk of FNSN. In this study, LTE was the most powerful predictor in multivariate analysis (Table 3). Nevertheless, the present study was a retrospective review of real-world data, and the patient populations were not selected or well designed, as in randomized clinical trials. Hence, the study power might be diminished because of patient/surgeon heterogeneity, though we did monitor these patients for more than 10 years. Our data provide real-world evidence regarding treatment practices for patients with FNSN.

The clinical significance of underestimating nodal staging has been discussed. Anderson et al. re-evaluated FFPE tissue blocks of SNs and found that 11% of patients had undiagnosed metastases in SNs during re-evaluation [23]. In the present study, four patients had cancer metastasis in nonrelevant SNs but were negative for metastasis in relevant SNs. Superselection of SNs as relevant or nonrelevant by surgeons is not necessary. All SNs identified with either methyl blue or radioactive compounds should be sent for frozen sectioning. Furthermore, metastasis in the internal mammary lymph nodes is possible in high-risk patients, and preoperative biopsy should be considered [24, 25]. We did not perform sentinel biopsy for the internal mammary lymph nodes, and underestimating nodal staging should be considered.

Sixteen other FNSN patients had results of benign lymphoid hyperplasia based on frozen sectioning of relevant SNs, whereas the final pathological report indicated metastatic carcinomas in FFPE blocks of the same nodes. The proportion of micrometastasis in SNs was seven of 20 FNSN patients. All patients with FNSN underwent delayed ALND. The survival rates of FNSN patients undergoing ALND and those without SN metastasis were similar (Fig. 2). The long-term recurrence rates were also the same (Table 4), and our results were consistent with those of other studies. Breast cancer patients with SN metastasis have survival outcomes similar to those without metastasis [6, 26]. For patients with early breast cancer and one or two SNs containing metastasis, 10-year OS with SLNB is noninferior to ALND [7]. Indeed, SLNB successfully replaces ALND in early breast cancer [27].

The present study included patients from 2005 to 2009 before the publication of the ACOSOG Z0011 trial results [6], and this is why ALND was performed for our FNSN patients. The two-step operation with delayed ALND has similar long-term morbidity but a longer operative time [28, 29]. The number of lymph nodes identified is slightly reduced in delayed ALND patients, but without clinical significance, and the risk of lymphedema is similar between delayed and immediate ALND [30, 31]. The major risk of delayed ALND for FNSN patients is perioperative and anesthesia-related distress, especially in elderly patients [32, 33]. In the present study, two patients had metastasis in SNs and nonsentinel ALNs. These two patients benefited from delayed ALND and had good survival outcomes. According to the results of the present study and the ACOSOG Z0011 trial, delayed ALND is unnecessary for patients with SN micrometastasis who are undergoing partial mastectomy. However, delayed ALND should be considered for those with SN macrometastasis after evaluating the risks of a secondary operation.

The limitations of the present study were the small number of patients and data missing due to preoperative assessments in other hospitals. Further evaluation of different subtypes of breast cancer according to a previous study [34] was difficult because of the small number of patients. Recent studies show that the sensitivity of SLNB can be improved by contrast-enhanced ultrasound or carbon nanoparticle suspensions [35–37]. However, our hospital does not have these capacities. In addition, the pathologist in our hospital only performed frozen sectioning for sentinel nodes without a cytological study of touch imprints, and artifacts from fast cooling can cause false interpretations of frozen sections. This was a retrospective study, and we collected data from chart reviews. The findings provide real-world evidence on treatment practices for patients with FNSN.

## Conclusions

SLNB is the standard approach for the axillary region in patients with early breast cancer and a clinically negative node. We conducted a case-matched control study. The incidence of FNSN was 6% in patients undergoing SLNB. Risk factors included a larger tumor size in preoperative mammography and an increased ratio of LTE in CNB. All 20 FNSN patients underwent a secondary operation with ALND. Our results demonstrate decreased sensitivity for intraoperative frozen sections in SN micrometastasis. Metastasis in nonrelevant SNs was detected for 4 of 20 FNSN patients, and two other patients showed metastasis in nonsentinel ALNs. The long-term survival of patients with FNSN after completing ALND was noninferior to those without metastasis in SNs.

## Data Availability

The raw data of this manuscript are available upon reasonable request from the corresponding author.

## Abbreviations

AJCC TNM stage: American Joint Committee on Cancer tumor–node–metastasis stage
ALN: axillary lymph node
ALND: axillary lymph node dissection
BCSS: breast cancer-specific survival
BCT: breast-conserving therapy
BI-RADS: Breast imaging reporting and data system
CI: confidence interval
CNB: core needle biopsy
DFS: disease-free survival
ER: estrogen receptor
FFPE: formalin-fixed paraffin-embedded
FNA: fine-needle aspiration
FNSN: False-negative frozen section of sentinel nodes
H&E: hematoxylin and eosin
HER2: Human epidermal growth factor receptor 2
HmR: Hormone receptor
HR: Hazard ratio
LTE: lymphatic tumor emboli
OCT: Optimal Cutting Temperature Compound
OR: odds ratios
OS: overall survival
PR: progesterone receptor
SN: sentinel node
SLNB: sentinel lymph node biopsy
TNBC: triple-negative breast cancer
